# The Treatment of Internal Hæmorrhoids by Means of Interstitial Injections of Carbolic Acid

**Published:** 1910-02-19

**Authors:** Dudley D'A. Wright


### Hospital Clinics.

### THE TREATMENT OF INTERNAL HÆMORRHOIDS BY MEANS OF INTERSTITIAL INJECTIONS OF CARBOLIC ACID.

By DUDLEY D'A. WRIGHT, F.R.C.S.

Although the method of treating internal hæmorrhoids by means of interstitial injections of solutions of carbolic acid is by no means new, its adoption and acceptance as a recognised or satisfactory procedure has not, as far as this country is concerned, been very widespread. In America—the land of its origin—it has been used by many practitioners, and has gained for itself considerable repute. It is true, however, that at its inception it was much abused by unqualified persons, who, without a knowledge of the anatomical or pathological elements concerned, pursued wholesale methods of injections, with the pure acid, or very strong solutions of it, in various, and often totally unsuited, rectal diseases. It is scarcely surprising that serious complications occasionally followed such procedures, and inevitably caused the method to fall into disrepute, a stigma from which its brilliant results in suitably chosen cases have not yet succeeded in freeing it.

### Some Essential Precautions.

For complete success of this treatment two essentials have to be kept in view: First, that it be applied to internal hæmorrhoids only; secondly, that the proper strength of the solution of the acid be used.

With regard to the first precaution, those piles which originate above the sphincter, whether they be prolapsed or not, are the only kind suitable. External hæmorrhoids cannot be treated by this method. Injection into them causes much pain and swelling, and may easily provoke abscesses, and eventually a fistula. But the true internal hæmorrhoid, whether of the bleeding type or not, is most amenable to treatment; and from an experience extending over many years, I must say that I have not met a case of this kind, however aggravated, which does not rapidly respond to the injections.

As to the second item. The recognised strength of the acid which hits off the happy mean is a 10 per cent. solution. I have always used the following mixture: Equal parts of extract of hamamelis and distilled water, to which is added carbolic acid to a strength of 10 per cent. This mixture was originally used by Dr. Eugene Hoyt, of New York, and it was that experienced surgeon who introduced this method to my notice fifteen years ago. I believe that the action of this mixture containing the astringent and styptic properties of the wych hazel is more certain and rapid than the simple solution of the acid in water. So far as I can gather from the published statements of the British surgeons who have used this method, their custom has been to employ a far greater degree of concentration of the acid than that which the experience of American surgeons has dictated, and to this I attribute their half-hearted advocacy of this form of treatment.

The great advantage of this method is that it is possible to carry it through from beginning to end without the necessity of the patient lying up, or having the daily routine of work interfered with in any way; and my experience leads me to think that to use strong solutions, even in the most suitable cases, is to put to the hazard a successful issue. Furthermore, I believe it is only under these circumstances that such complications as wide-spread thrombosis or suppuration—the bugbears which seem to loom so large in the eyes of many surgeons, and which deter them from making use of the method—can possibly occur; and I can assert that in Dr. Hoyt's hands throughout a period of over thirty years, and in my own smaller experience, the use of the weaker solution has not once been followed by either of these undesirable results.

It is essential that the hypodermic syringe be of such a type that it is easily sterilisable. Either there must be needles which can be discarded or boiled after being used, or else, which the writer prefers, those made of platinum, or other metal which can be sterilised by heating in the flame of a spirit lamp. It will be convenient to have one or more needles about 1¾ inches long so as easily to reach the piles when the speculum is used.

### Details of Examination.

The rectal speculum may be either the simple cylinder type—such as Howard Kelly's "Sphincteroscope," or cone-shaped with a portion of its wall removed and replaced by a sliding piece. For certain cases this is useful, as it permits of isolated areas of the mucosa being treated without other parts coming into view and obscuring the field. The slide can be withdrawn as far as is needed, and just so much of the piles allowed to present through the aperture as may be necessary. Bryant's modification of Hilton's speculum answers these requirements well. The ordinary bivalve is not a convenient speculum for this work, as it allows too much of the rectal wall, and especially of the perianal skin, to fall into the opening between the blades, thus obscuring the parts higher up, and interfering with the necessary manipulations. Mops of absorbent wool on holders are necessary to cleanse the walls of the bowel from mucus, fæces, etc., which might obscure the field of action.

It is, of course, necessary that the parts should be treated under good illumination. For this purpose there may be either a hand lamp which can be placed in proper position without the necessity of the operator holding it, or a small head lamp can be used. It is desirable that the patient should have a good action of the bowels before an injection is given; and when the first examination is made it is a good plan to give a hot-water enema and get the patient to evacuate it, and at the same time to strain, in order to bring the hæmorrhoidal tumours outside the sphincter. By these means we can gauge the extent of the trouble, and the amount of treatment required. In some cases this is not necessary, as the piles may be always readily brought outside by straining, or even by the patient standing erect.

So long as we are able to bring the hæmorrhoids into view the treatment may be carried on without the use of a speculum. Each tumour which presents may be injected with 3 to 5 minims of the solution, the smallest being treated first, as the swelling caused by the injection will make the larger piles obscure the smaller. The puncture should be made into the pile well away from the margin of the skin, and the needle should not penetrate into the tissues outside. This can be prevented if its entrance and passage into the swelling be made in a direction parallel with the bowel wall. The diffusion of the fluid into the interstices of the tumour usually causes a blanching of the surface which is very characteristic, though the absence of this blanching does not necessarily imply misapplication. The needle should not be withdrawn immediately after the injection, otherwise much of the fluid will escape through the puncture; time should be left for this to spread into the tissues of the pile. At first it is wisest not to inject all the masses which are visible, but to do two or three only; and after each injection it is absolutely necessary to replace all the prolapsed bowel and piles well above the internal sphincter, and to warn the patient to return them should they again come down, otherwise they will become rapidly swollen and may cause much trouble and pain.

If the above directions be carried out, the application is in many cases quite painless. In very irritable subjects some pain is complained of, but of no great duration. It needs a considerable amount of practice to hit off the exact depth of penetration, and consequently pain is more often the result of a beginner's efforts than of one who has had much experience. Patients vary much in the sensitiveness of the bowel, and some will feel no discomfort from an injection of even ten minims into a pile.

### After Treatment.

After a few treatments the piles will no longer stay out sufficiently to enable the injections to be given. Here, and in all cases which from the first do not prolapse sufficiently to enable the piles to come really clear of the sphincter, the speculum should be used. I would even advise a beginner to use a speculum in all cases except the very worst, for there is always a risk, in injecting piles which are outside, of puncturing them too near the skin margin. The result of this is to cause extravasation beneath the skin, and a painful swelling of the parts which takes several days to decline. The sphincteroscope having been inserted, the piles fall into its lumen as it is withdrawn, and when they are sufficiently in view the speculum is held steady with the left hand and the injection made with the right, holding the syringe between the first and second finger supported by the thumb, and pushing the piston home with the palm of the hand. The patient should always lie on the side for these injections. If the knee-elbow position be taken, the larger piles will not appear properly in the speculum, but will fall away from the operator. Anyone can satisfy himself that this is so by passing the speculum when the patient is reclining and seeing how much comes into view, and then examining in the knee-elbow position. He will be surprised to see how much falls out of view in the latter position.

Having made the injections, the piles are gently wiped free of blood or mucus by the wool on the holders, and the speculum withdrawn. If the slide speculum be used, only one pile at a time will be seen and injected, and it is usually necessary to remove and reintroduce it several times in one sitting to complete the injections. For this reason I prefer to use the sphincteroscope, which needs only one introduction, reserving the slide instrument for exceptional cases, or only towards the end of the case when a single pile needs treatment.

### Effects of Injections.

The injections are usually given twice weekly. though in certain cases three times a week may be quite safe. It is astonishing how quickly the symptoms are ameliorated. After the first few treatments patients often experience great relief, and in cases when moderate-sized hæmorrhoids prolapse outside the bowel whenever the patient goes to stool three or four injections will usually get rid of the trouble. But it should not be presumed that the patient is cured. Inspection by means of a speculum will show the presence of piles above the sphincter, and treatment must be continued until these have disappeared.

Whilst the treatment is proceeding it is necessary that the patient should have a daily action of the bowels and maintain a soft consistency of the motions to prevent any necessity for straining at stool. Some mild laxative should be taken to ensure this if necessary. Moreover, it is best if the patient exercises great moderation in the taking of alcoholic stimulants whilst under treatment; indeed, all patients who are subject to hæmorrhoidal affections will find their health improve if they abstain from alcohol. In addition to this it is as well that a large meal should not be taken immediately after an injection, as it is often the cause of discomfort; if the injection be given in the morning only a light luncheon should be taken. Apart from these no other alterations in the routine of daily life are necessary.

### Complications.

Ill-effects after the injections are very seldom met with. Pain may be complained of, lasting from half an hour to several hours; it is rarely acute, being more often a sense of fulness or heat in the parts, and if really troublesome may be allayed by bathing the region with hot water or applications of hot fomentations, and if severe a morphia suppository may be used. Swelling from extravasation of the injected fluid under the peri-anal skin will result in much tumefaction and pain, and can be avoided by making the injection well away from the anal margin, or "white line" of Hilton. Should, however, these complications occur, the patient should rest up and put on lead and opium lotion compresses. The bowels should also be kept inactive for two or three days.

Another complication I have seen occasionally arise is the appearance of a small fissure of the anal margin, caused, I think, by the erosive action of the injected fluid leaking out of the puncture and acting on some previously weakened spot in the anal canal. This gives the usual symptoms of fissure, pain coming on some time after defæcation and lasting for some hours. Fortunately this kind of fissure readily heals if the bowels be confined for three days and some hamamelis and cocaine ointment be supplied to the part night and morning.

Lastly, should the patient allow the piles to become extruded and leave them prolapsed, turgescence from the congested blood flow may lead to their strangulation. This should be prevented by replacing the tumours above the sphincter as quickly as possible. The swollen condition of the parts may render this a difficult matter if the piles have been allowed to remain outside a long time, but under these conditions assistance can be obtained by applying to the swelling a piece of lint soaked in adrenalin solution, and at the end of five minutes their bulk will have much diminished, and after being smeared with vaseline reduction will not usually be difficult. This complication is an easily preventable one if the patient be warned to replace the piles directly they protrude, and I have but rarely met with it in my practice.

In conclusion, I would reaffirm my conviction that the dangers and risks which are said to be run in having recourse to this form of treatment can be eliminated by using weak solutions, taking the precautions set forth in the foregoing remarks, and dealing with cases of internal hæmorrhoids only. When these lines are followed the treatment is as successful as the other operative measures, has the advantage of them in that the patient is able to be about during the treatment, and is freed from the unpleasant association of general anæsthesia.

